# Age-at-Death Estimation of Fetuses and Infants in Forensic Anthropology: A New “Coupling” Method to Detect Biases Due to Altered Growth Trajectories

**DOI:** 10.3390/biology11020200

**Published:** 2022-01-27

**Authors:** Mélissa Niel, Kathia Chaumoître, Pascal Adalian

**Affiliations:** 1 Aix Marseille Univ, CNRS, EFS, ADES, 13007 Marseille, France; kathia.chaumoitre@univ-amu.fr (K.C.); pascal.adalian@univ-amu.fr (P.A.); 2 Service de Radiologie et Imagerie Médicale, Hôpital Nord, CHU Marseille, Assistance Publique des Hôpitaux de Marseille, 13015 Marseille, France

**Keywords:** forensic anthropology, age estimation, femur length, *pars basilaris* shape, inverse Fourier transform, geometric morphometrics

## Abstract

**Simple Summary:**

In forensic anthropology, estimating the age-at-death of young juvenile skeletons is crucial as a direct determinant of legal issues in many countries. Most methods published for this purpose are based on either maturation or growth processes (two essential components of development) and focus on “normal” (i.e., nonpathological) growth. However, when the osseous remains available for study are from an individual that experienced an altered growth process, age estimation may be biased, and accounting for this would be helpful for potentially avoiding inaccuracies in estimation. In this research, we developed a method based on the combined evaluation of both maturation and growth. Maturation is evaluated by the conformation of the *pars basilaris*, a bone at the skull base that provides an indirect estimate of brain maturation, while growth is assessed using femoral biometry. The method was tested on two medical validation samples of normal and pathological individuals. The results show that it was possible to identify “uncoupling” between maturation and growth in 22.8% of the pathological individuals. Highlighting potential uncoupling is therefore an essential step in assessing the confidence of an age estimate, and its presence should lead experts to be cautious in their conclusions in court.

**Abstract:**

The coupling between maturation and growth in the age estimation of young individuals with altered growth processes was analyzed in this study, whereby the age was determined using a geometric morphometrics method. A medical sample comprising 223 fetuses and infants was used to establish the method. The *pars basilaris* shapes, quantified by elliptic Fourier analysis, were grouped into consensus stages to characterize the maturation process along increasing age groups. Each *pars basilaris* maturation stage was “coupled” to biometry by defining an associated femur length range. The method was tested on a validation sample of 42 normal individuals and a pathological sample of 114 individuals whose pathologies were medically assessed. Couplings were present in 90.48% of the normal sample and 77.19% of the pathological sample. The method was able to detect “uncoupling” (i.e., possibly altered growth) in more than 22.8% of samples, even if there was no visible traces of pathology on bones in most cases. In conclusion, experts should be warned that living conditions may cause alterations in the development of young individuals in terms of uncoupling, and that the age-at-death estimation based on long bone biometry could be biased. In a forensic context, when age has been estimated in cases where uncoupling is present, experts should be careful to take potential inaccuracies into account when forming their conclusions.

## 1. Introduction

Estimating an individual’s age-at-death from skeletal remains is one of the major issues in biological and forensic anthropology when assessing a biological profile. In the case of young individual skeletons, age-at-death is crucial to any analysis of biological remains. In forensic anthropology, a fetus’s legal personality is dependent on fetal age estimation, with the resulting social, ethical, and economic consequences [1], and the assessment of fetal viability and legislation on abortion and infanticide are also directly dependent on fetal and infant age estimation—hence contributing to the need and importance of developing reliable and accurate methods.

Several fetal and infant age-at-death estimation methods have been established. Most of these are osteometric, radiographic, or ultrasound methods [2,3,4,5,6,7,8,9,10,11,12,13,14,15,16,17,18,19,20]. They can be development based, which aim to estimate physiological age based on maturation processes (e.g., skeletal morphology, appearance and maturation of secondary ossification centers, maturation of dental germs), or biometric based, which rely on growth processes (e.g., crown–rump length, cranial and abdominal perimeters, and the maximum length of long bones).

However, the question of living conditions and, therefore, the context in which the development of a young juvenile took place can remain unanswered. Most methods assume that these conditions are “favorable” or “normal”, though these can obviously be disturbed by any pathological conditions experienced by the mother or child. In other words, the ontogenetic trajectory—the child’s developmental trajectory—is likely to be altered.

It is generally accepted that brain maturation is the best criterion to establish physiological age during early development, regardless of the environmental or socioeconomic conditions, even in cases of fetal or maternal pathologies [21,22,23]. The brain unfortunately undergoes rapid autolysis after death (within approximately 48 h) and can no longer be studied, but it has an influence on skull base osseous structures [24,25,26,27,28,29,30,31]. Therefore, these structures can be considered to be indirect and taphonomically resistant testaments of brain maturation.

To establish a biometric age, it is accepted that femoral length is the most reliable and accurate estimation indicator [3,7,8,9,32]. Nevertheless, growth-based age estimation may be biased in cases of growth delay or growth advancement caused by pathological conditions. These conditions are difficult to detect because most pathologies leave little or no trace on fetal and infant bones. Sherwood et al. [32] demonstrated that diseases causing abnormally short femurs (such as trisomy 21 or Turner syndrome) or abnormally long femurs (such as *spina bifida*) can lead to inaccuracies of up to almost four weeks in fetuses when estimating age at death.

Therefore, when only using femoral length without considering possible alterations in developmental conditions, one cannot know whether the age at death will be underestimated, correct, or overestimated with respect to the chronological age (real age).

Our biological hypothesis is that the physiological age (maturation) is more reliable and stable than the biometric age (growth), and that these two “different kinds of ages” are coupled for nonpathological individuals. Accepting this hypothesis, it can be argued that living conditions, whether they are simply “changing” or truly “unfavorable” to development, influence biometric growth more than maturation.

This “coupling” or agreement between maturation and growth processes could be used to assess and control fetal and infant age-at-death estimation, targeting individuals with growth variation due to possible pathological conditions. As a consequence, the demonstration of the “uncoupling” of these two processes would be an indication, or even serve as an alert, that the accuracy of the age-at-death estimation of a young juvenile skeleton must be considered with great caution.

As a direct indicator of skull base maturation and therefore an indirect indicator of brain (and thus general) maturation, we chose to use the *pars basilaris* of the future occipital bone [33,34,35,36]. We quantified its degree of maturation with geometric morphometric analyses from its outline shape. The estimation of biometric age (growth) was based on the maximum diaphyseal length of the femur.

These two bones are both dense and compact [11,37,38], and they are generally found to be in good preservation states considering forensic and archaeological contexts [11,37].

Using computerized tomography (CT) scan imaging of fetuses and infants with nonpathological conditions, the aim of our study was to develop a method based on the expected coupling between maturation and growth to detect possible growth variation.

Once established on a medical imaging sample (learning sample) of nonpathological individuals, the method was applied to a separate validation medical sample of nonpathological individuals and another sample of individuals whose pathologies were fully documented.

If an individual presents the “normal” (i.e., nonpathological) coupling variability established by the learning sample, the hypothesis of an alteration of his ontogenetic trajectory can be proposed. It is then necessary to discuss the potential reason for this alteration (growth delay or advancement in connection or otherwise with an identified pathology). Regardless, this study shows that estimated age must be considered with caution.

## 2. Materials and Methods

### 2.1. Sample

An anonymized database composed of 1136 individuals aged between 11 weeks in utero and 20 years old was compiled within UMR 7268 ADES (AMU-CNRS-EFS). From this, a medical imaging sample of 379 individuals aged 16 weeks in utero to approximately one and a half years (17.7 months) was derived.

#### 2.1.1. Normal and Pathological Development

**Figure 1:**
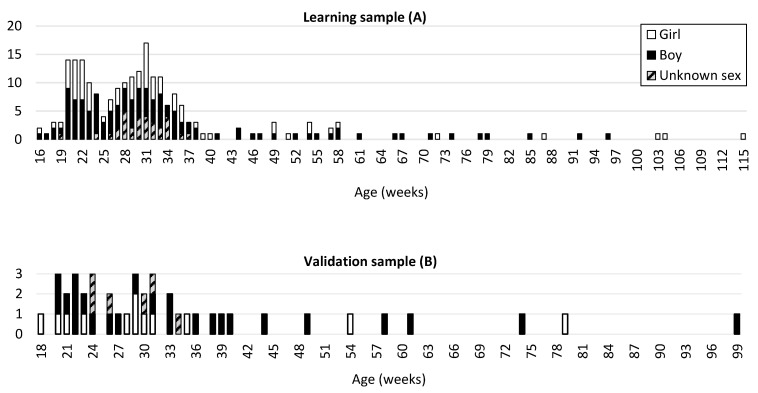

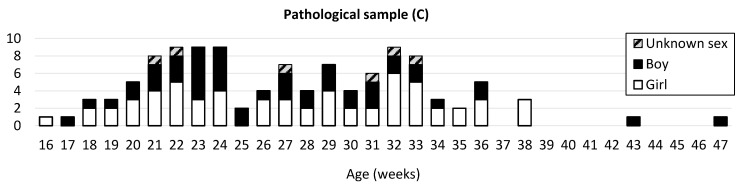
Age (in weeks) and sex distribution of the learning sample (**A**) comprising 223 individuals and the validation sample (**B**) comprising 42 individuals. Age (in weeks) and sex distribution of the pathological sample (**C**) comprising 114 individuals.

The studied population was divided into three samples. A learning sample (A) comprising 223 fetuses and infants with nonpathological conditions (77 girls, 115 boys, and 31 of unknown sex) ranging from 16 fetal weeks to 77 postnatal weeks (mean age: 33.28 fetal weeks; Figure 1) was used to establish the method. A second sample (B) comprising 42 fetuses and infants ranging between 18 fetal weeks and 61 postnatal weeks (mean age: 34.69 fetal weeks; Figure 1) was used as a separate validation sample. Given that the available age classes were not homogeneous for normal individuals, random selection by age classes was conducted to ensure a good representation of age; the selection comprised approximately 85% for the learning sample and 15% for the validation sample.

For our analyses, the ages of fetuses (based on accurate reports of the mother’s last normal menstrual period and ultrasound data obtained at 10 weeks of gestation, which is an obligatory examination under French law) and infants were expressed in weeks: from 16 to 38 weeks for fetuses and from 39 to 115 weeks for postnatal individuals. This means that a “45-week-old” individual is actually an individual aged 45 weeks minus 38 weeks (average length of pregnancy), which corresponds to 7 postnatal weeks.

Nonpathological conditions were essential for sample A and B individuals. The conditions considered for mothers were the absence of congenital disease, diabetes, or arterial hypertension. The nonpathological conditions of fetuses (such as the absence of external or visceral malformation, the absence of bone anomaly on a CT scan, the absence of cerebral anomaly on MRI, and normal karyotypes) were established based on multidisciplinary ante mortem and post mortem examinations conducted by medical experts of the prenatal diagnosis center. Concerning infants, CT scans allowed us to verify developmental normality. Examinations were performed in cases of road accidents, sudden or unexpected infant death syndrome, and forensic investigations.

Fetuses and infants with identified pathological conditions were included in a third sample (C) comprising 114 fetuses and infants (61 girls, 47 boys, and 6 of unknown sex) ranging from 16 fetal weeks to 47 postnatal weeks (mean age: 27.24 fetal weeks) (Figure 1C).

#### 2.1.2. Pathologies Groups

Depending on the pathological conditions, the following subgroups were established:

- Constitutional bone diseases or CBD (Ellis–van Creveld syndrome, thanatophoric dysplasia, achondroplasia, Jeune syndrome, facial femoral syndrome, VACTERL association, and harlequin ichthyosis = 14%);
- Growth disorders or conditions justifying differentiated growth or GD (intrauterine growth retardation, macrosomia/diabetes, and twin pregnancy = 39%);
- Localized anomalies or LA (skull, polymalformative syndrome, limbs, and spine = 23%);
- Cerebral anomalies or CA (21%);
- Chromosomal anomalies or CHRA (trisomy 21 and trisomy 18 = 3%).

The same individual could be classified in several types of pathologies, such as a localized anomaly and a cerebral anomaly.

### 2.2. Data Acquisition

The ante mortem and post mortem CT scans of sample A, B, and C individuals were collected from the Picture Archiving and Communication System (PACS) in the hospital of Marseilles (Assistance Publique—Hôpitaux de Marseille, France). Individuals were scanned using a helical CT scanner (Somatom Sensation Cardiac 64; Siemens, Erlangen, Germany). The scanning parameters were as follows: voltage of 100–140 kVp, amperage of 50–180 mAs, 512 × 512 pixels, resolution of 0.25–4.87 pixels per mm, voxel size of approximatively 0.5 × 0.5 × 0.6 or 1 mm^3^, and a slice thickness of 0.6–1 mm. These high-resolution native slices recorded in the Digital Imaging and Communications in Medicine (DICOM) format were anonymized before being used in the study, in accordance with the standards of the French National Consultative Ethics Committee for health and life sciences (CCNE) and the Helsinki Declaration of 1975 concerning the privacy and confidentiality of personal data.

### 2.3. Bone Reconstruction

Before reconstructing the femur and *pars basilaris* in three dimensions (3D), region of interest (ROI) segmentation on the DICOM slices was performed with the ImageJ^®^v1.51 software (National Institutes of Health, Bethesda, MD, USA) to separate the bone from adjacent tissues. The threshold value was obtained by calculating a threshold mean value (TMV) [38], which is an average of the half-maximum height (HMH) values [39]. The TMV was used in Avizo Standard Edition (v.7.0.0^®^, Visualization Sciences Group, SAS, Berlin, Germany) to reconstruct the 3D bone surfaces.

Since there are no significant differences between the right and left femur in young juveniles [3,9,10,40,41,42] and convention suggests that the left femur is preferred, we only measured the right femur when the left was not available.

### 2.4. Maturation Criterion: Elliptic Fourier Analysis of the Pars Basilaris

The complete protocol was described by Niel et al. [43] and was used in this study.

#### 2.4.1. Outline Process

Briefly, we defined a homologous reference plane for all the *pars basilaris* in the inferior (external) view. This was defined thanks to two type II and one type III landmarks [44]. Type II landmarks are the most posterior point of the left and right horns, and a type III landmark is the central point of the anterior surface. All landmarks were digitized on 3D reconstructed surfaces using Avizo Standard Edition^®^ software.

This step allowed us to project all reconstructions in the same 2D plane and with the same orientation. Then, outline shapes were quantified according to 150 equally linearly spaced points digitized along the *pars basilaris* contour with the tpsDig2 v.2.17^®^ digitization program [45]. Finally, the contour data of the *pars basilaris* were normalized using generalized Procrustes analysis (GPA) [46,47,48,49] based on four type II and III homologous landmarks [44] called control points [46].

#### 2.4.2. Measurement Error

Repeatability (intra-observer error) and reproducibility tests (inter-observer error) were realized to validate the protocol on 30 randomly selected individuals in sample A. Repeatability was tested by the same observer repeating the protocol twice several weeks apart; for reproducibility, a second observer applied the protocol once.

#### 2.4.3. Harmonics Number

With EFA, one may wonder what the appropriate number of harmonics is, since this number determines the accuracy of the contour reconstruction. The following two paragraphs of text is the explanation as reproduced from Niel et al. pp. 37–38 [43]:

According to the Nyquist theorem [50], the harmonic number must be less than half the number of sampled outline points. Consequently, on the 150 points sampled for EFA, only the first 74 harmonics were retained for analysis. Given that we cannot retain all the Fourier coefficients for our analysis” (74 harmonics × 4 coefficients = 296 coefficients), because the measurement error is expected to increase with harmonic ranks, the percentage of error on harmonic coefficients was calculated using a Procrustes analysis of variance (ANOVA) on the three sessions [51]. This procedure calculated the mean sums of squares for the four coefficients of each harmonic to observe the evolution of error according to the rank of the harmonics (in percentage). Only the first harmonics, showing an acceptable digitization error rate, were retained for further analyses. An error rate under 35% is considered to be reasonable in an outline analysis using EFA [51].

The assessment of the total percentage of measurement error was then performed using a Procrustes ANOVA [51,52,53,54,55] adapted to elliptic Fourier coefficients [51]. The Fourier coefficients of the coupled series are used in the Procrustes ANOVA with the number of harmonics previously defined. The intra- and interindividual variances were directly calculated from the means of the sums of squares and crossed products corresponding to individuals and residual sources of variation [51]. These residuals, representing the variability between the two sessions, correspond to the measurement error [55]. 

### 2.5. Coupling between Maturation and Growth Process

#### 2.5.1. Maturation Criterion: Shape Stages

Maturation stages were established on the *pars basilaris* shapes of the nonpathological learning sample A to visualize the *pars basilaris* morphological changes through time. With this sample, consensus shapes from 4 to 26 weeks with overlap every 2 to 13 weeks were created, which enabled us to have intermediate shapes and, thus, a continuous vision of maturation from 16 weeks in utero to 77 postnatal weeks. Thus, 19 stages of consensus shapes, defined by the mean of 5–52 shapes depending on stages, were obtained (Table 1). Then, to visualize and compare the morphology of each consensus shape, the *pars basilaris* outlines were reconstructed from Fourier coefficients with the inverse Fourier transform function [56,57,58].

#### 2.5.2. Growth Criterion: Femoral Lengths

Femoral diaphysis lengths were measured (in millimeters) on Avizo Standard Edition^®^. Percentiles were calculated from sample A according to each maturation stage of the *pars basilaris* and used as growth criteria (Table 1). To include a greater range, a margin of ten percentiles was added at each extreme, calculated as the difference between 0 and 10 percentiles and between 100 and 90 percentiles, thus providing 0–10 percentiles and 100 + 10 percentiles, respectively (Table 1).

### 2.6. Statistical and Morphometric Analyses

#### 2.6.1. Bilateral Femoral Asymmetry and Sex Effect on the Variables 

Between-sex comparisons of the *pars basilaris* shapes were explored using nonparametric multivariate ANOVA (MANOVA) [59], and the between-sex comparison of the femoral lengths was performed using Kruskal–Wallis rank sum testing. The bilateral femoral asymmetry was explored using a *t*-test.

#### 2.6.2. Application of the Coupling Method in Samples B and C

Each *pars basilaris* of samples B and C was tested, one at a time, by comparison with the 19 stages representing the maturation consensus shapes. Once the outlines were quantified with EFA after the GPA procedure, assigning a maturation stage to the tested *pars basilaris* was realized by calculating the Euclidian distance (or Procrustes distance) between the centroids of the 19 consensus stages and the tested (compared) shape [60,61]. The minimal distance between the centroid of the tested *pars basilaris* and one of the 19 consensus shapes allowed for the assignation of a stage to the *pars basilaris*.

For growth, the measurement of the tested individual femoral length was compared to the range expected for the defined maturation stage (Table 1). If this measurement was found to be within the expected range, we considered that growth corresponded to the maturation stage values and there was “coupling”. Then, it could be concluded that growth was “normal” (i.e., nonpathological). On the contrary, if growth did not correspond to the maturation stage values, then “uncoupling” had occurred.

Analyses were performed using *RStudio* (developed for R software—Version 1.1.383—^®^ 2009–2017 RStudio, Inc., Boston, United States) and the software packages *Momocs* [62], *Morpho* [63], *geomorph* [64], *car* [65], *gap* [66], *efourier*, and *iefourier* functions [56].

## 3. Results

### 3.1. Quantification of Pars Basilaris Shapes

#### 3.1.1. Number of Harmonics

**Figure 2:**
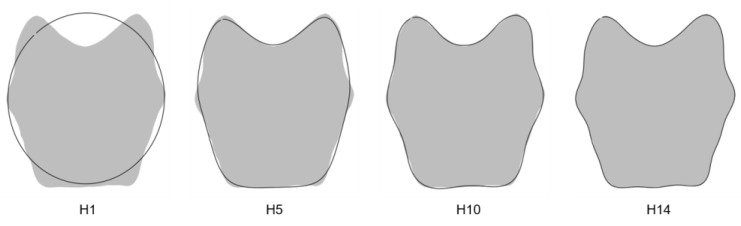
Outline reconstructions of the *pars basilaris* (dark outline): harmonics 1, 5, 10, and 14. Gray shapes represent the reconstruction of the *pars basilaris* with the maximum number of harmonics (74 in this study).

The percentage of measurement error was inferior to the threshold defined at 10% for the first 14 harmonics, corresponding to 56 Fourier coefficients per individual. This allowed us to faithfully reconstruct the outline of the *pars basilaris* (Figure 2).

#### 3.1.2. Measurement Error

The percentage of measurement error for the outline protocol is 1.13% for repeatability and 1.96% for reproducibility for the selected first 14 harmonics. This protocol is reliable and reproducible.

### 3.2. Between-Sex Differences and Femoral Length

The nonparametric MANOVA showed that there were no significant shape differences between sex groups (*F* = 1.503, d*f* = 2, *p* = 0.199) and the femoral lengths were not significantly different between sex groups (*p* = 0.706). Additionally, there was no bilateral asymmetry (*p* = 0.239) between the right and left femoral diaphysis.

### 3.3. Coupling between Maturation and Growth

**Figure 3:**
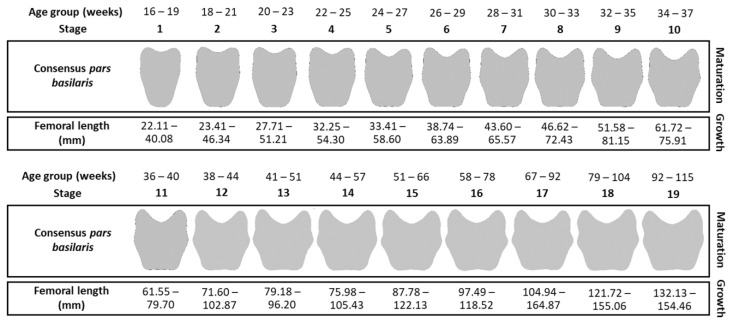
Maturation and growth criteria by stage and age group (in weeks).

The maturation and growth criteria are summarized in Figure 3. Each maturation stage corresponds to a range of femur lengths defined by the lower bound (0–10 percentiles) and the upper bound (100 + 10 percentiles), corresponding to the extremes.

#### Method Application

The method was applied to the validation sample B and the pathological sample C to verify whether growth and maturation were coupled or, in other words, whether the individual’s growth corresponded to the values expected by their maturation stage (Figure 3).

In sample B, we observed coupling in 90.48% of samples. The four cases where uncoupling was detected correspond to two growth delays (−4.82 and −1.70 mm) and two growth advancements (+1.26 and +13.13 mm). These values were calculated by subtracting the femoral length of the tested individual (*X^T^*) at the upper (*I^s^*) or lower (*I^i^*) values of the expected interval for the maturation stage, depending on whether individual measurement was inferior or superior to the interval.

For a measurement inferior to the interval:

*X^T^* − *I^i^* = −*x* or growth delay,

For a measurement superior to the interval:

*X^T^* − *I^s^* = *+x* or growth advancement.

In sample C, 26 individuals (22.81% of the sample) showed uncoupling. Most of them were girls (61.5%). Uncoupling in these cases corresponded to 14 cases of growth delay (from −23.02 to −1 mm) and 12 cases of growth advancement (from +0.43 to +6.61 mm).

**Figure 4:**
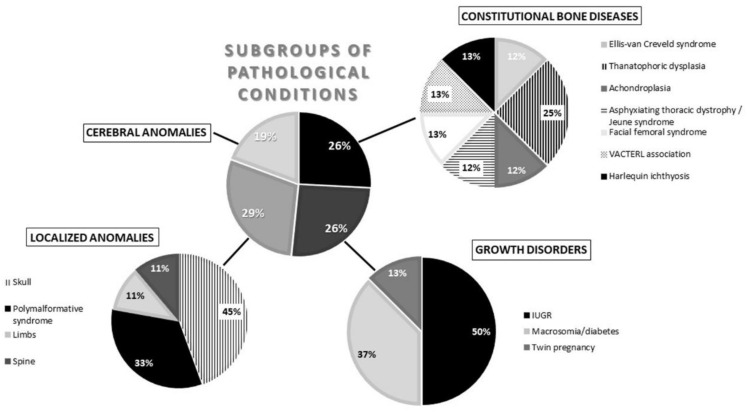
Chart summarizing the subgroups and the detailed pathological conditions for individuals in the medical imaging sample with uncoupling. IUGR = intrauterine growth retardation.

Regarding the subgroups of pathological conditions for uncoupling, LA was the most represented (29%), followed by CBD (26%) and GD (26%) in equal parts; CA was the least represented (19%). More precisely, individuals in the LA subgroup who were most likely to have uncoupling were those presenting a cranial anomaly (45%), followed by polymalformative syndromes (33%) and limb anomalies (11%). In the GD subgroup, IUGR was the most common pathology (50%), followed by macrosomia/diabetes (37%). Then, among individuals with CBD, uncoupling was more frequently observed for the thanatophoric dysplasia cases (25%) and in relatively equal parts for the other diseases. Finally, CA was the least frequent in uncoupled individuals (19%) (Figure 4).

## 4. Discussion

### 4.1. The Fetus and Infant Sample

In France, since the advent of prenatal diagnosis centers (Decree 97–578 of 28 May 1997, consolidated on 11 May 2018, France), fetuses have been systematically examined in cases of medically interrupted pregnancy or spontaneous death (miscarriages and in utero deaths). A panel of experts’ analyses medical records follows ante mortem (CT scan in utero) and post mortem (complete visceral examination, histological study, fetal karyotype, placenta examination, description of external and visceral abnormalities, and front and profile radiography) examinations. After respecting a strict anonymization protocol, we could access these examinations records and be informed about malformations (bone or visceral), chromosomal abnormalities, or even the precise determination of the cause of death.

For sudden and unexpected infant death and forensic cases, CT scans and autopsies are performed only with the written consent of the parents. Not all parents agreed, which is why there were few available exams. Moreover, sudden and unexpected infant death generally occurs before the age of one year according to the High Authority for Health, which stated in its 2007 report that 80% of sudden infant deaths occur before the age of 6 months, with a peak at 2–3 months. This is consistent with the age distribution of our study sample.

For children aged more than 1 year, we could access some rare autopsy reports and some ante mortem CT scans, which are mostly performed for infants who have fallen or have been in a car accident. Cases are rather rare, and when they exist, the whole body is rarely examined to avoid unnecessary radiation. For our analyses, however, we required images that at least included the portion of the body from the skull base to the proximal end of the tibia. All of these elements made it difficult for us to obtain a large sample of fetuses and infants and almost impossible to have homogeneous age groups.

The second difficulty in studying young individuals concerns the CT scan quality. We first sorted the CT scans according to their image quality as excellent, average, or poor. This sorting forced us to rule out many examinations that were not exploitable for our study (due to flowing bone surfaces, incomplete bone, and irregular contours). It should be recalled that fetal X-ray exposure increases the risk of malformation (teratogenic effects) and long-term cancer induction (carcinogenic effects) [67], so the dose of radiation should be as low as possible.

In the case of a postmortem CT scan, the dose of radiation may be higher because the same ethical concerns are no longer relevant. These obtained slices were generally of high or excellent quality and therefore represent the largest part of our studied material.

### 4.2. Quantification of Shape

Geometric morphometric methods and EFA have already been used to quantify the *pars basilaris* shape changes and intrastage variability during the second and third trimesters of fetal life [43]. EFA is suitable for considering the curved morphology and small thickness of this bone, since it is difficult to digitize homologous landmarks on the surface. This difficulty, combined with the fact that the only definable landmarks are not linked to the overall object geometry, oriented us toward a mathematical description of the outline to analyze the global shape of the *pars basilaris.*

As explained by Niel et al., 2019 (pp. 40–41) [43], outline analysis (and, more specifically, Fourier descriptors) provide complex and detailed information regarding the shape. Additionally, this method has been frequently used for discriminating biological forms quantifying morphological differences [46,51,57,58,68,69,70,71,72,73,74,75], as the use of ellipses means that the shape description in EFA is global and therefore helpful for describing bones with curved edges [70,76]. This indicates that it is perfectly suited for characterizing the morphology of the *pars basilaris*.

In the development of the method, a few available landmarks were used to define the reference plane and normalize the Fourier descriptors. The normalization of the control point using GPA [46] prevents the homology problems encountered in specimen alignment on the major axis of the first ellipse, which is conventionally used for the normalization of Fourier descriptors [77]. This method was not adapted to *pars basilaris* because the ratio between the length and width changes as the child develops [11,18,37,78,79]. It has also been shown that among the various normalization methods, the one using the control point with GPA is the most appropriate to use for bones with a few homologous landmarks and circular contours [70,76], such as the *pars basilaris*.

### 4.3. Interest in the Pars Basilaris

Because of its early formation, between the 10 and 14 gestational weeks [11,78,79,80,81,82,83,84,85], and its robustness, the *pars basilaris* is one of the elements of the future adult occipital bone most used to establish age-at-death estimation methods for fetuses and infants. Methods using this bone generally use conventional morphometry and/or bone size ratio [11,15,18,37,78], but they do not consider the shape, which might be valuable in improving age estimation.

Thanks to geometric morphometric methods based on Cartesian landmark coordinates, some researchers have been interested in shape to document the skull base changes through development, though with no intention of age estimation. Shape is defined as the geometric properties of an object that are invariant to scale, rotation, and translation, whereas the form of an object includes both its shape and size [60,86] (Needham equation: form = shape + size) [87].

Transposed onto our biological or forensic anthropology context concerning bones, the shape corresponds to bone maturation and the size corresponds to growth. The advantage of geometric morphometric methods is their ability to precisely quantify and visualize morphological variation through powerful statistical tools [60,86]. Based on these methods, previous studies have described the fetal cranial base development as a whole [30,36,78,88,89], but the *pars basilaris* morphology has rarely been separately analyzed.

Moreover, most morphometric methods focus on a single anatomical area to estimate age. We believe that the multiplication of age estimators, in addition to increasing reliability and accuracy [90], would minimize estimation errors [32,78,91], an idea that is consistent with some previous studies [79,92,93]. For example, according to Tocheri and Molto [91], linear measurements of the *pars basilaris* make it possible to refine the estimated age according to the degree of dental eruption and the maximum length of the femoral diaphysis. 

Other studies have shown that femoral length coupled with histological study and the combination of several fetal measurements (biparietal diameter, head circumference, abdominal perimeter, and femur and radius length) improve the accuracy of fetal age estimation [92,93]. Additionally, the *pars basilaris* maximum length is significantly correlated with age, crown–rump length, and humerus length [94]. These studies demonstrate that it is possible to refine age estimation through the use of conventional morphometry together with a combination of several parameters.

### 4.4. Morphology of the Pars Basilaris

In the literature, several authors have used traditional morphometry to demonstrate that the *pars basilaris* dimensions evolve during fetal and infant development [18,23,78,79], and the bone characteristics intensify with age [23]. The morphological characteristics of the *pars basilaris* are used not only in anatomy but also in biological anthropology, as they can give an idea about the fetal and infant age [11,15,18,37,78,79].

Using geometric morphometric methods, shape analysis confirms the increase in morphological changes from 18 to 41 gestational weeks [43]. The conclusions of our own study allow researchers to precisely quantify and visualize shape changes of the whole *pars basilaris* during prenatal development and after birth for the first time.

By studying *pars basilaris* shapes, forensic anthropologists will gain a better idea of fetus or infant ages since each maturation stage is associated with an age interval. In addition, regarding the WHO definition of viability (more than 22 amenorrhea weeks) and the term of a pregnancy, maturation stages higher than 3 can indicate whether a fetus is viable, and stages 11 and 12 are helpful for marking the term of the pregnancy.

### 4.5. Maturation and Growth Criterion

In our method, femoral length was chosen as the growth criterion because of its strong relationship with age, and the *pars basilaris* shapes gathered in 19 consensus stages were used to characterize maturation. The grouping of shapes into stages based on consensus shapes with overlaps enable one to obtain a logical continuity of maturation for fetuses and infants while also allowing one to compensate for the low number of individuals of certain age groups.

Growth was defined according to the maturation stages, and we used percentiles, since we sometimes had few individuals per stage. As in any inferential approach based on population sampling and because we are aware that the variability in femur size is not limited to that observed in our samples, which were sometimes of limited size, we widened the range. For this, extreme percentiles were added to either side of the 0 and 100 percentiles. As with growth charts, the use of percentiles allows for growth to be precisely “quantified” with limited statistical bias. Thus, for a given stage, if the length of the femur is below or above the extreme percentiles, growth is considered to be altered.

### 4.6. The Two Main Advantages of This Coupling Method

The method established in this study makes it possible to analyze the link between the biometric (growth) and physiological (maturation) age of fetuses and infants by coupling the maturation process estimated by means of the *pars basilaris* outline and the growth process estimated by means of the femoral diaphyseal length.

The results obtained from the nonpathological validation sample (B) are encouraging for the fetus and infant age-at-death estimation. We reported coupling in 90.48% of samples, so not only can our method confirm the “overall normality” of this nonpathological sample (first advantage), but we can also be confident when using a method with femoral length to assess age (second advantage).

Only 4 out of the 42 individuals of sample B showed uncoupling, and they never exceeded a shift of two stages of *pars basilaris* maturation. According to medical reports, these individuals did not have any identified pathological conditions, but in addition to the variability that we tried to include as much as possible in our learning sample (A), several factors can explain uncoupling, such as parity [95,96,97,98,99], parent general height and build [100,101], and the overall progress of the pregnancy, including the exchanges between the fetus and the placenta [100,102,103,104,105,106,107,108,109,110]. These appear to just have a slightly different variability from our learning sample and confirm that no method can be expected to be 100% reliable due to normal human variability.

### 4.7. Pathological Uncoupling

As previously mentioned, age estimation from femoral length may be biased since the individual may have had abnormal growth [32], which is not necessarily visible at first sight. This is particularly true when there are no visible bone deformations or malformations such as those which can be seen on fetuses with thanatophoric dysplasia type I-II, osteogenesis imperfecta type IIA, hypophosphatasia, achondrogenesis type IA-II, or diastrophic dysplasia group) [111]. For example, a small stature is found in trisomy 21 fetuses, whose femoral lengths are smaller than normal [112,113] and there are no obvious bone deformations that alert about this pathological state. Additionally, various chromosomal abnormalities or chronic utero-vascular insufficiencies can bias estimations of fetal biometric age [32].

Disease-related bone conditions are not always visible on a skeleton because, for the lesions caused by these conditions to be visible, the individual must be immunologically affected enough to allow disease development yet strong enough to survive it [114]. For example, there are no visible traces on fetal or juvenile human osteological remains of individuals affected by plague, whooping cough, smallpox, measles, scarlet fever, or even osteomyelitis or congenital syphilis, since the disease causes death before any bony stigmas can develop. Thus, childhood disease is not obviously observable from a skeleton, especially when the skeleton is moderately preserved [37].

In our study, uncoupling concerns: localized anomalies, constitutional bone diseases, growth disorders, and cerebral anomalies. Cerebral anomalies are related to size anomalies and malformations: there is one case of cerebral hypotrophy, one case of cerebral gliosis, one case of hydrocephalus, one case of bilateral frontal paraventricular cysts, one case of infection with necrotizing and viro-induced malformative ventriculoencephalitis cytomegalovirus, and one case of agenesis of the corpus callosum associated with microcephaly. Constitutional bone diseases form a heterogeneous group of conditions responsible for insufficient stature or abnormalities in the structure of the bone, whether or not associated with deformities [115]. Among these, uncoupling indicated one case of achondroplasia, one case of Ellis–van Creveld syndrome, one case of Jeune syndrome (or asphyxiating thoracic dysplasia), two cases of thanatophoric dysplasia, one case of femoral-facial syndrome, one VACTERL-type association case, and one case of harlequin ichthyosis.

For all the affected individuals, the femur growth did not match *pars basilaris* maturation. Some authors have further stated that the femoral length is the most suitable biometric parameter for distinguishing bone dysplasias: fetuses with a femur below 30% the mean for gestational age would have achondroplasia; fetuses with a femur between 40% and 60% the mean for gestational age would have thanatophoric dysplasia or type II osteogenesis imperfecta; and fetuses with a femur below 80% the mean for gestational age would be affected by hypochondroplasia, achondroplasia, or type III osteogenesis imperfecta [116].

For uncoupling in individuals with growth disorders, two individuals were found to have diabetes, one macrosomia, four IUGR, and the last one had a twin pregnancy. All these abnormalities or simple variations in growth (twin pregnancy is not necessarily a pathological pregnancy) could lead to either growth delays or advancements depending on the description of the symptoms, evidence for which can be retrieved with this method.

However, not all individuals in our pathological sample showed systematic uncoupling since the growth disorders associated with each disease depend on several factors such as their origin, their arrival during pregnancy, and their severity. This is the reason why only a few cases were detected. For example, the severity of macrosomia varies according to maternal, pregestational, and gestational diabetes, regardless of association with obesity [117,118]. Macrosomia is also associated with the mother’s age (the more advanced, the higher the risk) and parity (the more pregnancies the mother has had, the greater the risk) [118]. Unfortunately, this information cannot be verified since it had not been entered into our database.

Regarding IUGR, a fetus will develop this condition if it cannot achieve its genetic potential for growth due to genetic or external phenomena modifying this potential, or because an abnormality during pregnancy causes growth restriction [119]. Again, the severity of IUGR depends on its cause, the timing of its occurrence during pregnancy, and the duration of the intrauterine aggression [119]. Generally, fetuses with IUGR catch up in terms of their height during the second year of life, often as early as one year [120,121,122]. A child over 3 years of age who has still not caught up to his height should be taken care of by a pediatrician endocrinologist for in-depth examinations on stature delay, with a view initiating growth hormone treatment from the age of four [121,122,123,124]. It should be added that in cases of IUGR, cerebral maturation is generally not affected [125,126].

Additionally, there are variations in growth for multiple pregnancies compared to single pregnancies. For twins, a difference in the mean weight for gestational age is noted from 30 weeks [119]. The differences in growth between twins can be explained by the type of pregnancy; if it is monochorial–biamniotic, the transfusion–transfused syndrome is the first explanation. In bichorium–biamniotic pregnancies, the difference can be explained by a malformation of one of the twins. Placental anomalies and poor fetoplacental exchanges (nutritional, hypoxic, or toxic) can also explain growth anomalies [119].

Finally, the uncoupling of individuals with one or more localized anomalies concern:

Skull anomalies in four cases:

- (1–2) Two microcephaly cases (one was associated with craniosynostosis);
- (3) One ossification defect of the vault with the enlargement of the fontanelles and the presence of Wormian bones in the parietal and occipital region;
- (4) One severe hydrocephalus;

An anomaly of the limbs for one case:

- Anomaly of the femurs with shortening and curving;

An anomaly of the spine for one case:

- A *spina bifida;*

Three cases of polymalformative syndrome:

- One case with arthrogryposis, club feet, clenched hands, 11 pairs of slender ribs and platyspondyly;
- One case with abnormalities of the spine and ribs, as well as retrognathism;
- One case with anomalies of the spine, a short thorax, and a malposition of the four limbs (clenched hands, knees in extension, and club feet).

Finally, the cases of uncoupling highlighted by our method suggest that when maturation and growth do not match, experts must be prepared for a possible anomaly or variation in growth that risks biasing the age as estimated from femoral length.

Thus, the proposed method should be used in forensic anthropology for age estimation to verify whether growth has been altered by possible pathological conditions. This appears to be crucial in forensic contexts, where age estimation should be as accurate as possible to assess viability, set at 22 weeks of amenorrhea or a weight of 500 g according to WHO recommendations, to determine whether an individual came to term and to provide an unbiased age-at-death for police investigations.

To improve this method in the future, it would be of interest to include more healthy individuals to reduce the age range for some stages in order to provide greater precision in determining the consensus shape. The inclusion of samples from various origins would also allow the method to be used in different populations, and it could also be used in a clinical setting for screening for abnormal growth.

## 5. Conclusions

This study was focused on characterizing the link between maturation and growth by analyzing bone shape and biometry. The use of geometric morphometric methods and elliptical Fourier analysis enabled us to precisely quantify the *pars basilaris* shape changes from 16 fetal weeks to approximately one and a half years (17.7 months) in an unprecedented way.

By considering the coupling between the maturation and growth process, it is possible to detect potential anomalies or variations in growth. It is important to remember that it is difficult to macroscopically detect bone anomalies that could alert one to this possible variation and that the application of age-at-death estimation methods can be biased since they were established from reference populations with normal development but that the targeted individuals do not necessarily meet this condition.

In cases of uncoupling, experts should be warned that living conditions have altered the development of a young individual and that the age-at-death estimation based on long bone biometry may be biased. In a forensic context, the detection of uncoupling must lead an expert to be careful in their conclusions regarding the age determined for a young juvenile.

## Figures and Tables

**Table 1:** Sample A: age group (in weeks), number of weeks, and number of individuals according to the 19 *pars basilaris* maturation stages as well as femoral growth in percentiles (minimal values of 0–10, 10, 50, and 90 and maximal value of 100 + 10, in millimeters).

Stage	Age Group (Weeks)	Number of Weeks	Number of Individuals	Percentiles				
				0–10	10	50	90	100 + 10
1	16–19	4	9	22.11	25.57	30.09	35.33	40.08
2	18–21	4	34	23.41	31.58	37.93	41.12	46.34
3	20–23	4	52	27.71	34.73	39.42	44.36	51.21
4	22–25	4	36	32.25	36.88	43.19	47.18	54.30
5	24–27	4	28	33.41	43.17	49.34	54.97	58.60
6	26–29	4	37	38.74	48.04	52.20	56.72	63.89
7	28–31	4	50	43.60	51.15	56.70	60.42	65.57
8	30–33	4	51	46.62	56.22	59.28	62.54	72.43
9	32–35	4	36	51.58	58.09	62.53	66.44	81.15
10	34–37	4	23	61.72	63.59	65.56	71.68	75.91
11	36–40	5	14	61.55	65.66	71.14	76.48	79.70
12	38–44	7	8	71.60	73.41	77.71	88.29	102.87
13	41–51	11	9	79.18	82.26	90.68	94.95	96.20
14	44–57	14	15	75.98	85.46	94.79	104.14	105.43
15	51–66	16	13	87.78	94.42	103.51	111.75	122.13
16	58–78	21	10	97.49	107.55	112.40	117.30	118.52
17	67–92	26	9	104.94	111.99	117.91	135.63	164.87
18	79–104	26	7	121.72	123.99	132.02	151.45	155.06
19	92–115	24	5	131.92	132.13	149.79	152.05	154.46

## Data Availability

As mentioned in the informed consent signed by the mothers, the data for individuals will remain strictly confidential.

## Abbreviations

CA	Cerebral Anomalies
CBD	Constitutional Bone Diseases
CHRA	Chromosomal Anomalies
CT scan	Computerized Tomography Scan
DICOM	Digital Imaging and Communications in Medicine
EFA	Elliptical Fourier Analysis
GD	Growth Disorders
GPA	Generalized Procrustes Analysis
IUGR	Intrauterine Growth Retardation
LA	Localized Anomalies
MANOVA	Multivariate Analysis Of Variance
MRI	Magnetic Resonance Imaging
VACTERL	Vertebral, Anal, Cardiac, Tracheal, Esophageal, Renal, and Limb
WHO	World Health Organization
